# Sialolithiasis of the Submandibular Gland: Report of Cases

**DOI:** 10.7759/cureus.4180

**Published:** 2019-03-06

**Authors:** Sheik P Arifa, Pradeep J Christopher, Senthil Kumar, Srivatsa Kengasubbiah, Vandana Shenoy

**Affiliations:** 1 Oral and Maxillofacial Surgery, Thai Moogambigai Dental College & Hospital, Chennai, IND

**Keywords:** sialolithiasis, salivary gland disorder, submandibular gland sialolithiasis, submandibular gland disorder, sialolith

## Abstract

Sialolithiasis is the formation of calcific concretions within the parenchyma or ductal system of the major or minor salivary glands, but it most commonly affects the submandibular salivary gland. Sialolithiasis usually occurs in adults aged 30 to 60 years and causes pathognomonic pain during meals. The treatment of sialolithiasis depends on the size and location of the calculi. We present two cases of sialolithiasis of the submandibular gland managed via the intraoral and extraoral approaches, depending on the position of the calculi.

## Introduction

The submandibular gland (a major salivary gland) is a mixed, predominantly mucous gland with a large superficial section and small, deep lobes that connect around the posterior border of the mylohyoid muscle at the angle of the jaw [1]. The submandibular duct arises from the deep part of the gland from the floor of the mouth along the lateral side of the frenulum linguae [2].

Wharton’s duct rests at the lower level of the oral cavity, and this location allows for retrograde infection of the gland by oral flora. The pH of saliva in the submandibular gland is alkaline [3], which may lead to the formation of calcium salts [4]. The actual etiology of sialolithiasis is unknown. Clinically, the calculi appear as generally yellowish, round or oval masses and can be rough or smooth. Here, we discuss two cases of submandibular duct sialolithiasis.

## Case presentation

Case 1

**Figure 1:**
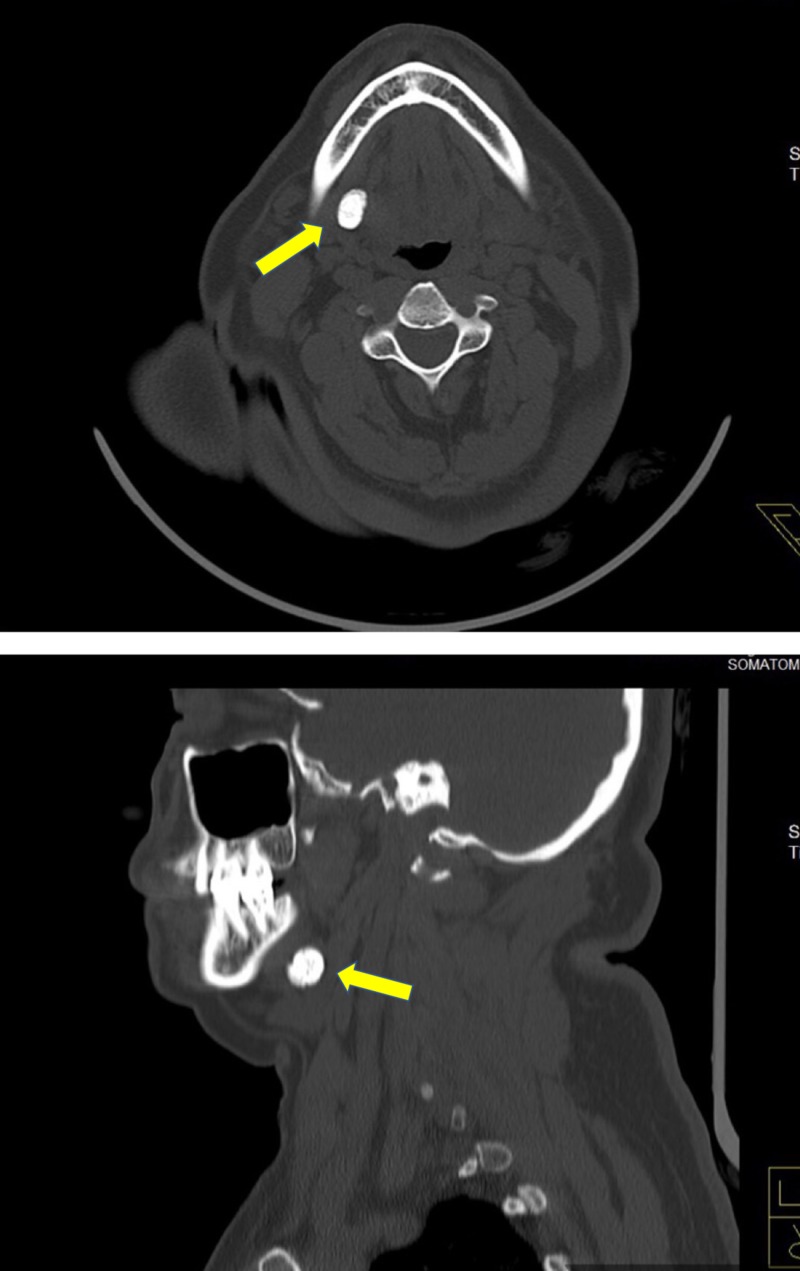
Axial and sagittal view of computed tomography showing sialolith

**Figure 2:**
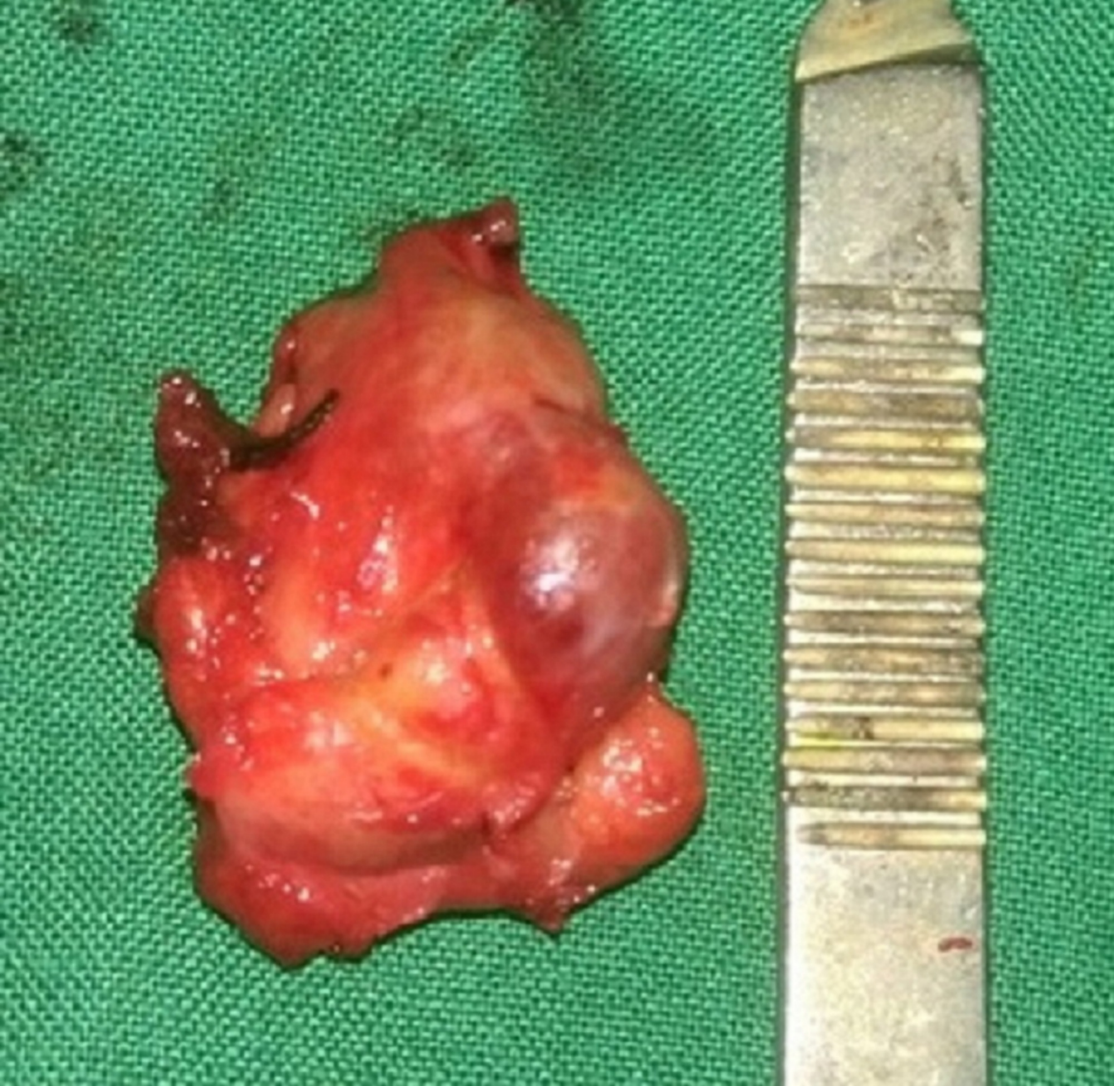
Excised submandibular salivary gland

A 64-year-old female patient reported with pain and swelling in her right submandibular region, noting the pain had persisted for 10 days. She reported concerns of intermittent swelling and pain during meals, which resolved after the meal, and she reported unpleasant sensations while eating sour or acidic food. On clinical examination, we noted swelling on her right submandibular region, extraorally. There was no discharge associated with the swelling. Bimanual palpation revealed a firm swelling in the posterior floor of the mouth. We clinically diagnosed the patient as having right submandibular salivary gland sialolithiasis. The diagnosis was confirmed by computed tomography (CT) (Figure 1). The process of surgically removing the gland began with a Risdon incision placed 3 cm to 4 cm below the mandible. We identified and protected the marginal mandibular nerve and then removed the submandibular gland from the mylohyoid muscle. We then divided and ligated the submandibular duct to remove the gland along with the calculi, which was an approximately 7 mm spherical formation (Figure 2). We placed a suction drain and closed the wound in layers. The patient experienced no postoperative complications on follow-up evaluations.

Case 2 

**Figure 3:**
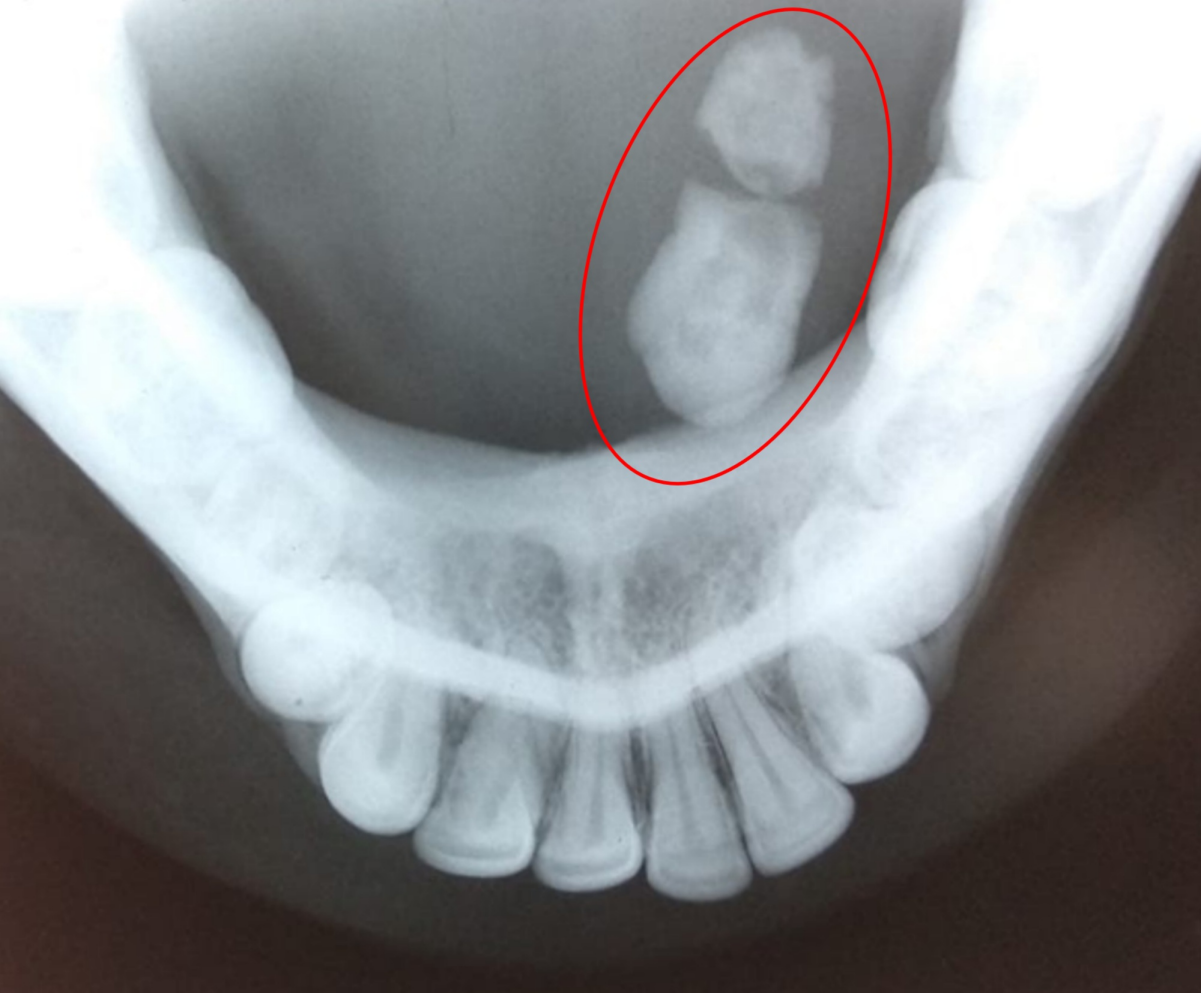
Occlusal radiograph showing salivary gland calculi

**Figure 4:**
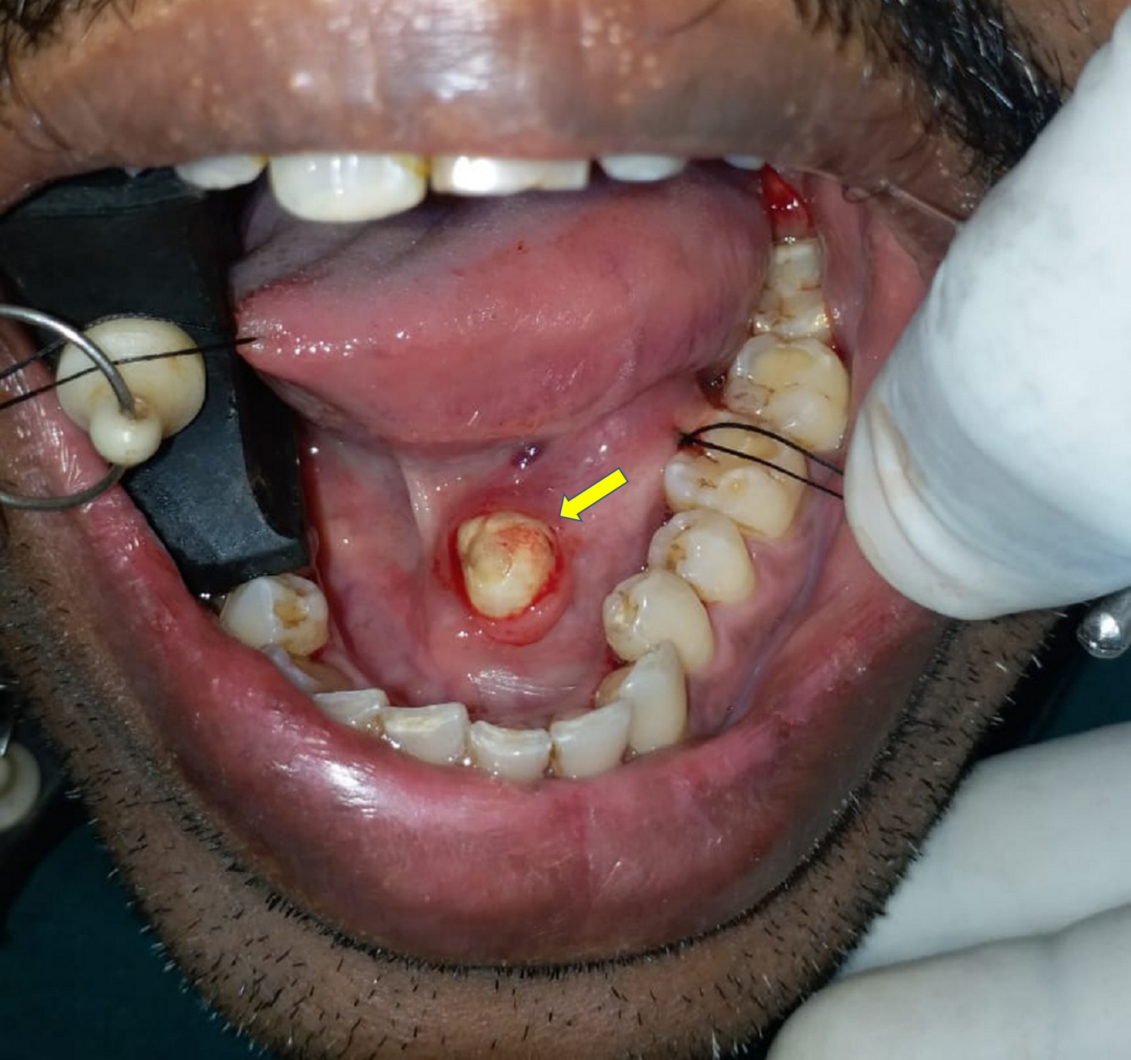
Intra-oral view

**Figure 5:**
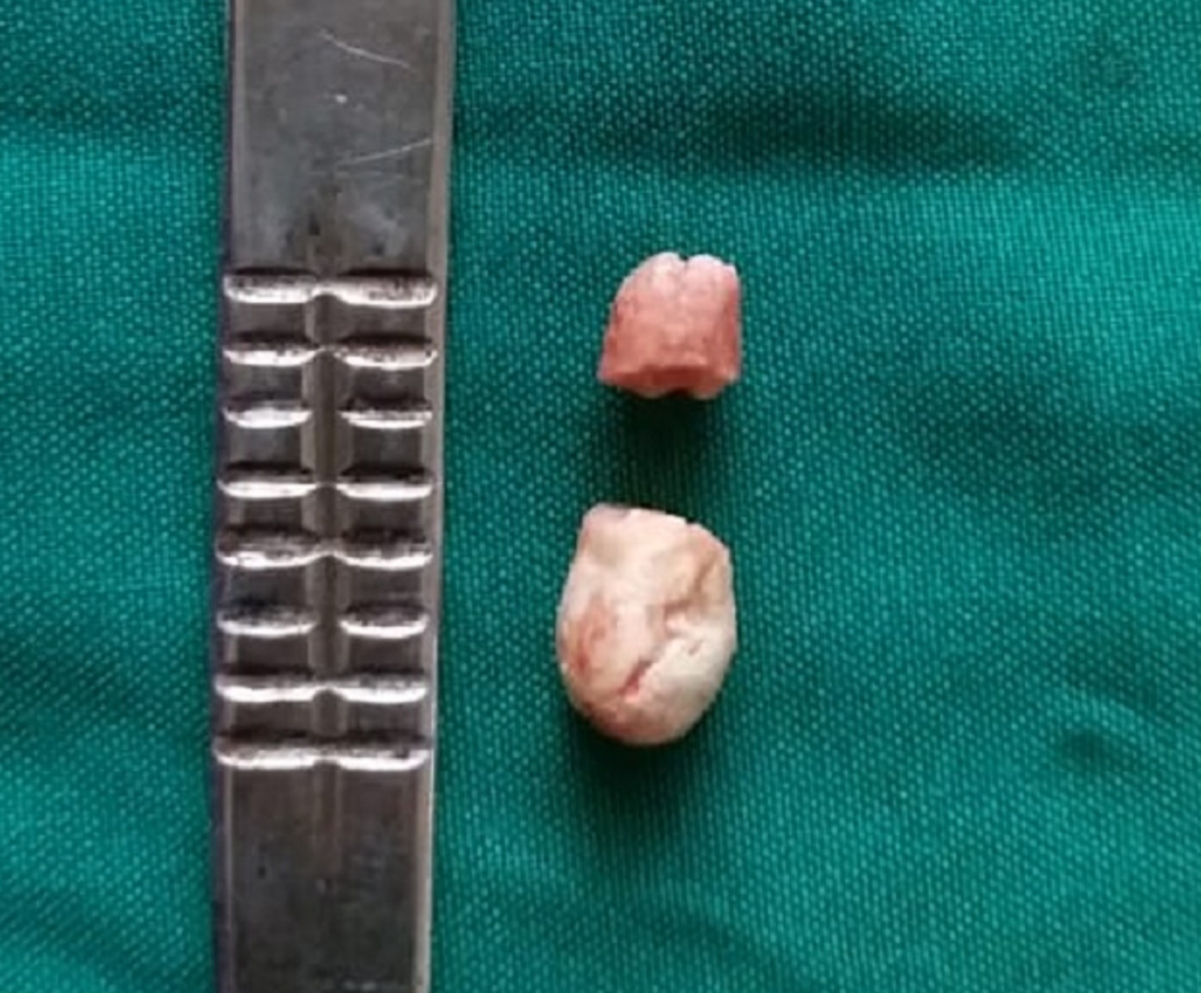
Submandibular gland sialolith

A 36-year-old male patient reported with swelling below the tongue and pain during meals. On examination, we palpated a hard swelling on the left submandibular gland duct. The swelling was confirmed by occlusal radiograph showing radio-opacity medial to 46,47 (Figure 3). We diagnosed the swelling as left submandibular salivary gland duct sialolithiasis. While the patient was under local anesthesia, we placed a stay suture to prevent the stone from gliding posteriorly. We then placed an incision over the stone to expose the calculi and facilitate its removal (Figure 4). We sutured the duct at the level of the mucosa on the floor of the mouth. The calculus was roughly oval and measured 10.4 mm (Figure 5). The patient experienced no postoperative complications on follow-up evaluations.

## Discussion

The submandibular gland lies within the submandibular triangle formed anteriorly and posteriorly by the digastrics muscle and inferiorly by the mandible. The gland has a large superficial section and a small deep lobe. Wharton’s duct exits on the medial surface of the gland. Sialolithiasis is the formation of calcific concretions in the major or minor salivary glands [5]. Sialolithiasis is more prevalent in men than in women and is rarely found in children. Most cases (75%) of sialolithiasis occur on one side of the gland, 3% occur bilaterally, and 1.2% of cases will atrophy. The gland most commonly affected is the submandibular gland (80% to 92% of cases) followed by the parotid gland (6% to 20%) and the sublingual and minor salivary glands (1% to 2% of cases).

There is no proper etiology for sialolith formation. However, reports suggest that intracellular micro calculi formation acts as a base for the stone formation. Dyschylia and increasing microlith formation lead to an increased level of bacteria, which then leads to focal obstruction and atrophy of acinar cells, ultimately causing secretary disturbances. Another theory attributes the formation to an increase in calcium and phosphate salts [6], which, in turn, form a deposition along with desquamated cells, salivary mucus, and bacteria. Other potential causes for sialolithiasis are infections, salivary dysfunction, ductal anomalies, foreign bodies, and ductal epithelium metaplasia. The ability of the calculi to grow to excessive size depends mainly on how the affected duct reacts.

The submandibular gland is the most common site of sialolithiasis because of its mucous, alkaline nature combined with the presence of a tortuous duct [7]. With improper salivary secretion, saliva stasis can lead to infection over time. Even with no infection, long-term salivary stasis can cause gland atrophy and fibrosis [8].

Swelling and pain are the cardinal signs of sialolithiasis, and proper diagnosis depends on taking adequate patient history along with a clinical evaluation [9]. X-ray imaging can help in the diagnosis, but smaller or hypomineralized calculi can only be found via other radiographic methods like sialography, ultrasound [10], CT, magnetic resonance imaging (MRI), scintigraphy, and sialoendoscopy.

Sialography is a technique to detect salivary gland calculi and visualize the whole duct system. Sialography is not indicated in acute infections or for patients sensible to contrast medium. Sialography is not advisable if a radiopaque calculus is found in the distal portion of the duct because the injected contrast medium could move the calculus nearer the gland and complicate its removal [3]. When sialography is not indicated in a case of suspected sialolithiasis, scintigraphy could be used. CT scans are not as invasive as sialography and, therefore, is the mode of choice for detecting sialoliths. A recently developed way to directly visualize sialoliths within ducts is sialoendoscopy-a new method that mitigates conventional radiology in the event of suspected salivary obstruction [3].

Treatment modalities, such as extracorporeal short-wave lithotripsy and sialoendoscopy, are effective alternatives to conventional surgical excision for smaller sialoliths. For large sialoliths, transoral sialolithotomy with sialodochoplasty or sialadenectomy remains the main method of management.

The differential diagnoses involve a radiolucent phlebolith, dystrophic calcification of the lymph nodes (with a cauliflower-like appearance), palatine tonsilliths(multiple and punctuate), and hemangiomas with calcification.

Once removed, health care providers should advise patients to adopt a diet rich in proteins, liquids, and acidic foods to prevent the formation of new sialoliths in the salivary gland [3].

## Conclusions

Sialolithiasis is a common salivary gland disorder, especially for the submandibular gland. Preoperative history and clinical and radiographic examinations are crucial for establishing the clinical diagnosis and treatment protocol.
